# A Case of Neuropsychiatric Systemic Lupus Erythematosus With Hair Loss as the First Diagnostic Symptom

**DOI:** 10.3389/fpsyt.2022.839566

**Published:** 2022-05-13

**Authors:** Xun He, Xi-Ling Duan, Jing-Song Liu

**Affiliations:** ^1^Department of Dermatology, Sichuan Academy of Medical Sciences, Sichuan Provincial People's Hospital, Chengdu, China; ^2^Department of Head and Neck Surgery, Sichuan Cancer Hospital, Chengdu, China

**Keywords:** neuropsychiatric systemic lupus erythematosus, case report, hair loss, diagnosis, symptom

## Abstract

Neuropsychiatric systemic lupus erythematosus is a serious complication of systemic lupus erythematosus. A 33-year-old female patient had repeated hair loss for more than 3 years. A dermatologic examination showed several pieces of irregularly shaped hair loss patterns in the center of the patient's scalp. The systemic treatment included oral hydroxychloroquine, aspirin enteric-coated tablets and prednisone, and intrathecal injection of dexamethasone and methotrexate. The local treatment included intralesional injection of triamcinolone acetonide and lidocaine in the lesion area, 0.1% tacrolimus ointment for external use. After 2-month treatment, hair regrew in a non-scarring patchy alopecia region with no further hair loss.

## Introduction

Neuropsychiatric systemic lupus erythematosus (NPSLE) is a complication of systemic lupus erythematosus (SLE) with an incidence rate of 21–95%. It has become the main cause of death in the activity period of SLE (1, 2). The diagnosis and treatment of NPSLE are challenging due to its complicated manifestations (3, 4).

According to the American College of Rheumatology, twelve central nervous systems (CNS) symptoms and seven peripheral nervous systems (PNS) symptoms are related to NPSLE (5). Lupus psychosis is an uncommon CNS symptom that usually occurs at the early stage of SLE. The occurrence of lupus psychosis is closely related to CNS damage caused by immune disorder. Metabolic disorders and glucocorticoid therapy may also be involved in the pathogenesis of NPSLE.

The diagnosis of NPSLE mainly includes the detection of autoantibodies, cerebrospinal fluid analysis, electrophysiological studies, neuropsychological assessment, and neuroimaging detection. However, none of the above methods has been accepted as a gold diagnosis standard. A previous study showed that the positive rate of serum antiphospholipid (APL) antibodies was significantly higher in patients with NPSLE than that in other patients with SLE (6). Thus, the level of APL antibody should be routinely examined in patients with SLE with neuropsychiatric symptoms. The cerebrospinal fluid (CSF) examination is the most basic examination in NPSLE diagnosis with a positive rate of 91.4%. EEG is used to study the onset of NPSLE, which detects the abnormalities, including asymmetrical brain electrical activity disorder, diffused background activity, and focal epileptic discharges (7). Neuroimaging examination is also critical for the early diagnosis of NPSLE. The initial onset of active lupus encephalopathy usually shows abnormities in diffusion-weighted imaging (DWI) and fluid-attenuated inversion recovery (FLAIR) (8).

## Case Presentation

A 33-year-old female patient with repeated alopecia for over 3 years visited our hospital in August 2015. Initially, a piece of alopecia area with an irregular shape of a broad bean size was inadvertently found on the top of the head. There were no obvious itches, rashes, or pain in the alopecia areas and their surroundings. The patient was diagnosed with “alopecia areata” in a local hospital, treated with medication (details were unknown), and the condition was improved. However, the alopecia recurred later, and the patient occasionally went to a local hospital for treatment. The diagnosis was ibid, but the curative effect was poor. The area of alopecia had a gradually expanding trend. More than 1 month ago, the area of alopecia rapidly expanded based on the original alopecia with no obvious incentives. The patient felt no other discomfort and visited our hospital. Her appetite, stool, and urine were fair, and her sleep was poor. The medical history of this patient showed that she suffered from schizophrenia 10 years ago and was treated by long-term oral administration of risperidone. The mental condition of the patient was stable. The affected area did not have a history of trauma. None of her family members had a similar disease. The patient had a childbearing history 5 years ago, and the spouse and child were healthy. There were no obvious abnormalities in the menstrual cycle and its amount. The consent from the patient for the publication of the case was obtained.

**Figure 1:**
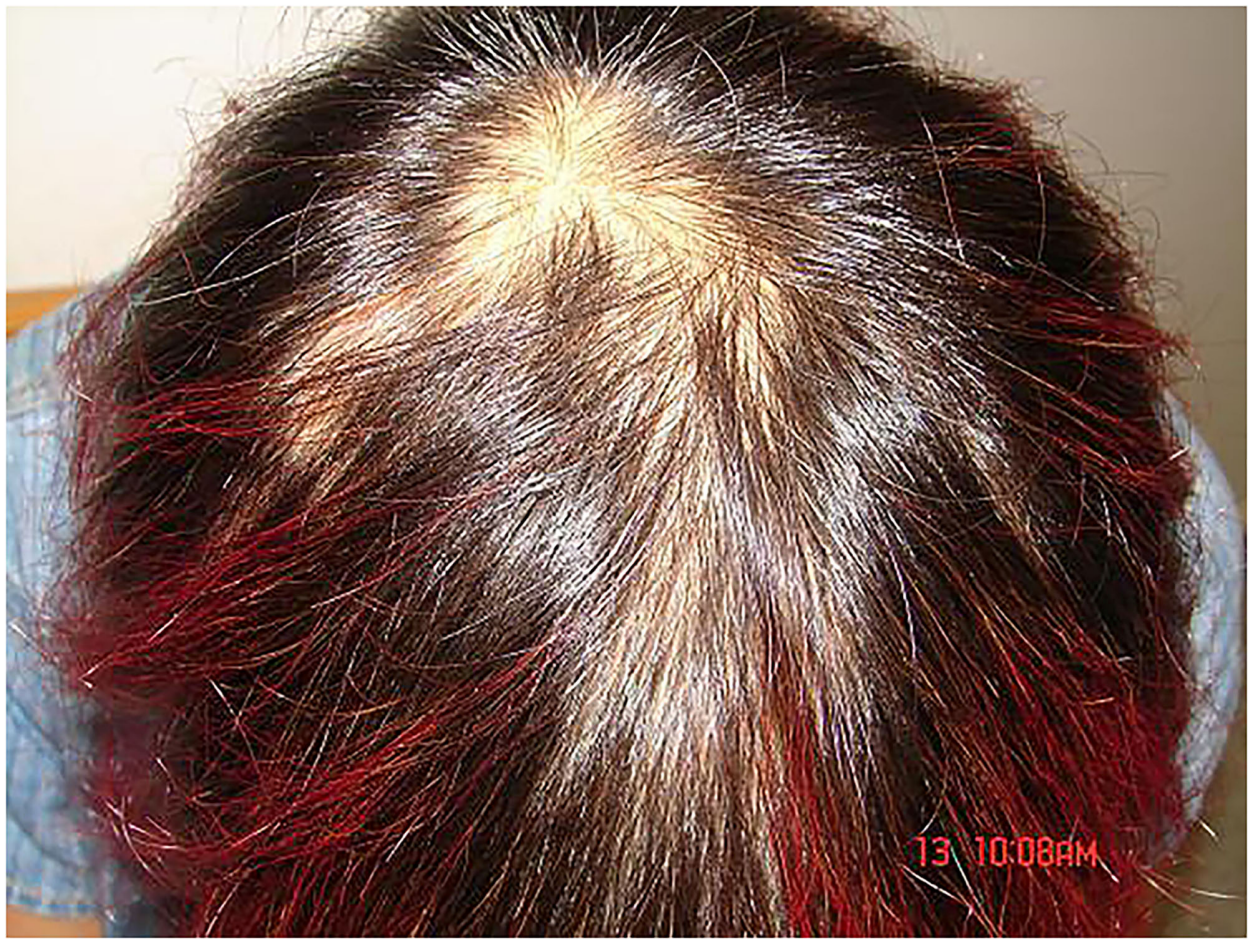
With the head as the center, multiple areas of alopecia with irregular shape.

**Figure 2:**
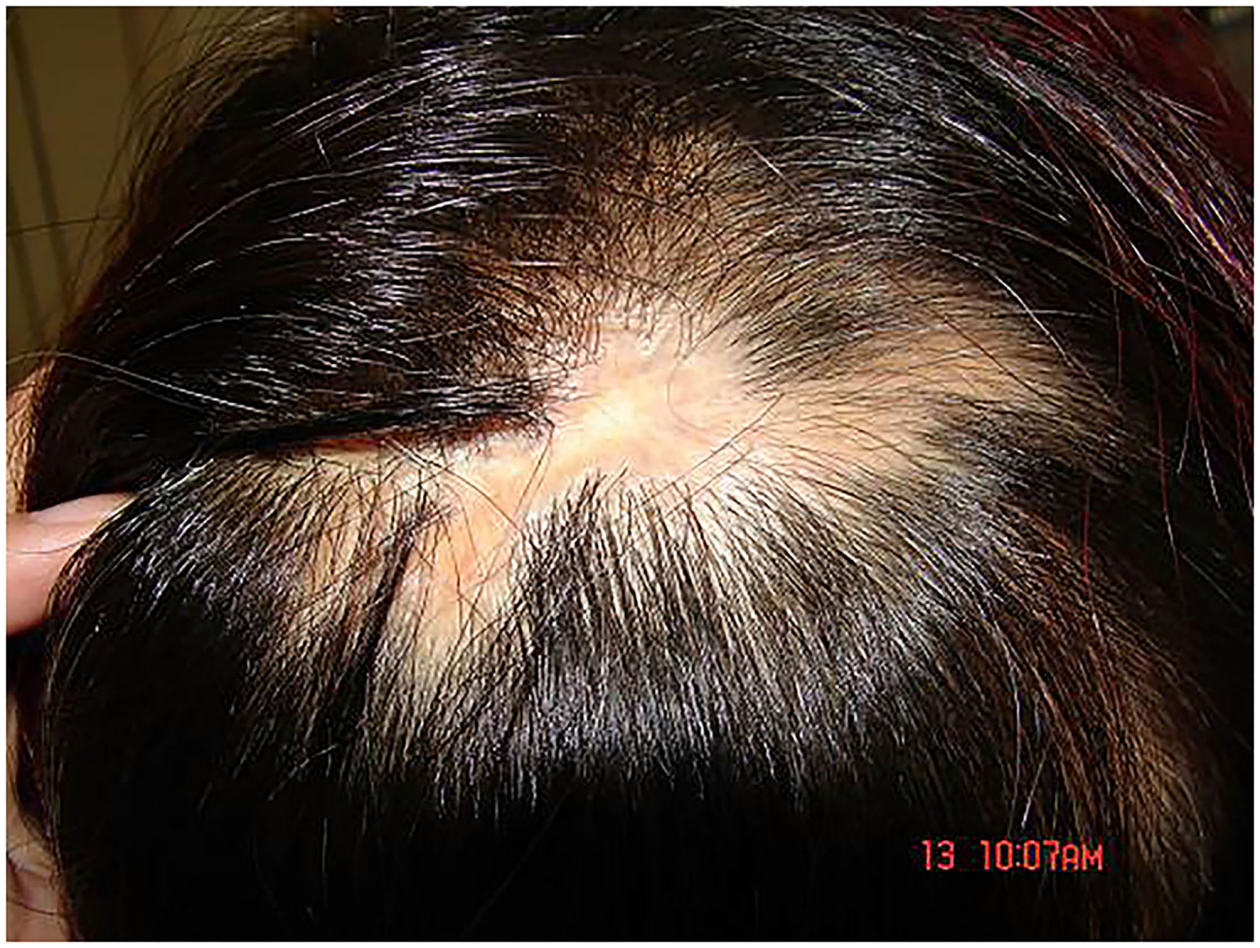
Obviously stripped atrophic scars were found in the alopecia area from the top of the head to the occiput posterior.

**Figure 3:**
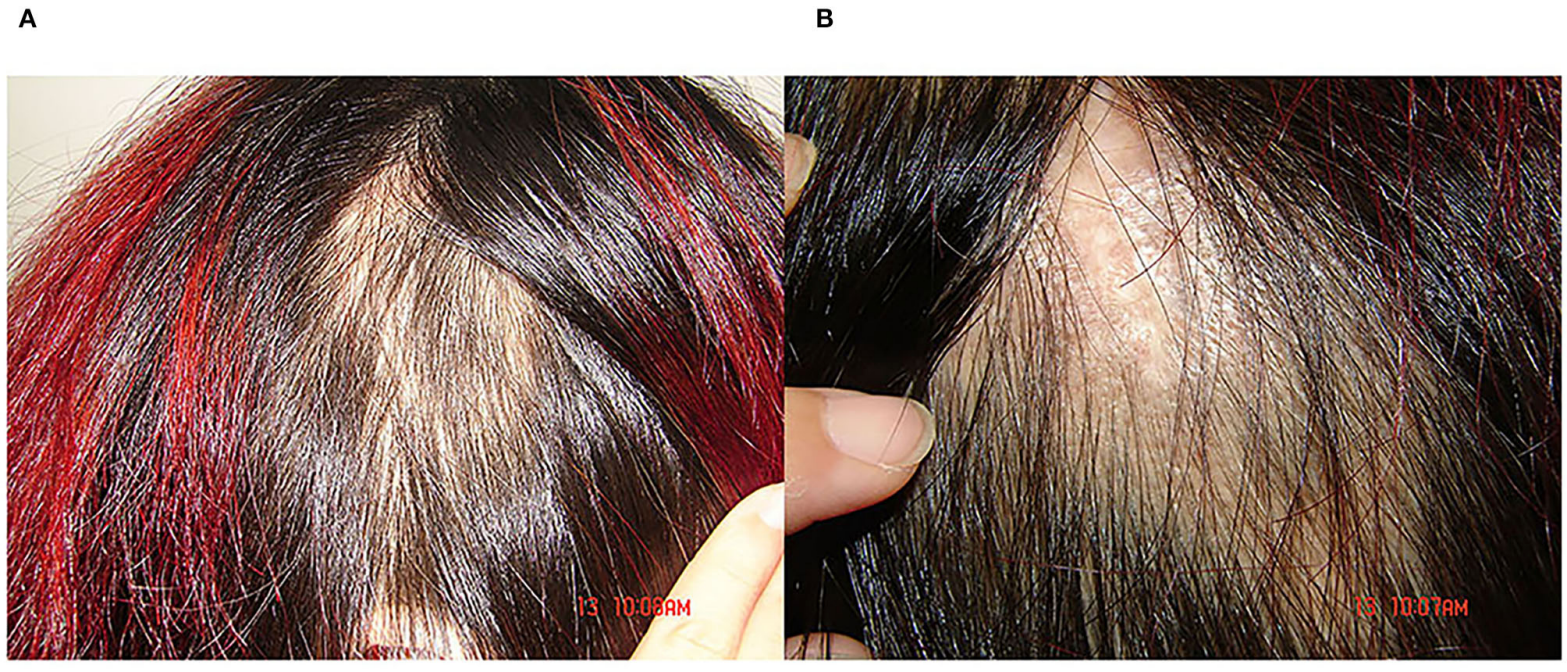
**(A,B)** Light erythema was visible in the new alopecia area, hair growth with a normal appearance could be observed within the alopecia area, the follicular orifice remained, and no obvious perifollicular keratosis, scales, broken hair, and exclamation mark-like hair were found.

Physical examination: no abnormalities were found. Conditions of dermatology: multiple areas of alopecia with irregular shapes were involved in the double temporal sides, parts were fused (Figure 1), and stripped atrophic scars were found in the alopecia area from the top of the head to the occiput posterior (Figure 2). Light erythema was visible in the new alopecia area. Hair grew with a normal appearance within the alopecia area. The follicular orifice remained. No obvious perifollicular keratosis, scales, broken hair, and exclamation mark-like hair were found (Figures 3A,B). Laboratory and auxiliary examinations: WBC count was 3.4 × 10 E9/L and PLT count was 87 × 10 E9/L. The urine test was normal. No abnormalities were found in liver and kidney function and electrolytes. The levels of immunoglobulin A (IgA), immunoglobulin (IgG), immunoglobulin M (IgM), and C4 were normal. C3 concentration, erythrocyte sedimentation rate (ESR), and anti-nuclear antibody (ANA) were 0.4026 g/L, 36 mm/H, and 1:320, respectively. The anti-ds-DNA and ACA were positive (+). Cerebrospinal fluid (CSF) pressure was 250 mmH_2_O, and protein concentration was 0.6 g/L. No abnormalities were found in the number of cells, glucose, and chloride. MRI results showed multiple diffused abnormal signals in the bilateral cerebral hemisphere and basal ganglia. No abnormalities were found in Chest X-ray, electrocardiogram, and abdominal color Doppler.

### Diagnosis

Systemic lupus erythematosus complicated with neuropsychiatric systemic lupus erythematosus.

### Treatment

Oral administration of hydroxychloroquine, 200 mg, bid; aspirin enteric coated tablets 200 mg, QD; prednisone 60 mg, QD, administrated for 3 consecutive weeks. The dose was reduced when the disease was under control, every 10 mg was reduced per 1–2 weeks, and the dose was reduced to 30 mg in 2 months, QD. Dexamethasone and methotrexone were each 10 mg intrathecal injected once weekly for two weeks. Triamcinolone acetonide 50 mg combined with lidocaine hydrochloride 5 ml were intralesional injected once every two weeks for four times; and 0.1% tacrolimus was applied outside, BID. At the same time, the patient was ordered to avoid sun exposure, and prevent fatigue and infection. 2 months post treatment, no further alopecia was found, and new hair grew in the non-cicatricial patchy alopecia area. The WBC, PLT, C3, and ESR values were 8.6 × 10 E9/L, 101 × 10 E9/L, 0.9301 g/l, and 13 mm/H, respectively. Cerebrospinal fluid pressure was 146 mmH_2_O. Protein concentration was 0.56 g/l. No abnormalities were found in cell number, glucose, and chloride.

## Discussion

Alopecia is a common clinical manifestation of SLE with an incidence rate of 17.3–54% (9). Alopecia in SLE is categorized into six types: telogen effluvium, non-cicatricial patch alopecia, lupus hair, the female pattern of alopecia, DLE pattern of alopecia, anagen effluvium, and mixed pattern of alopecia (10). The DLE pattern of alopecia belongs to the cicatricial alopecia, while the others are non-cicatricial alopecia. This case, reported in this study, had both the DLE pattern of alopecia and the non-cicatricial patch alopecia. It has been reported that the disease activity in patients with mixed patterns of alopecia was higher than that in patients with simple or diffused alopecia (11).

In SLE alopecia areata, the scalp may be pale red or red and the follicular orifice is well-preserved. The typical dermoscopic signs in alopecia areata, such as exclamation-like hair, broken hair, and black spot signs, as well as the infiltration of thronged-like lymphocytic around anagen follicles, are not observed in SLE alopecia areata (12).

The life quality and mental health scores were lower in patients with SEL with NPSLE compared to the ones without alopecia. Even after the systemic disease has been relieved, alopecia might still compromise the patient's self-esteem and leads to long-term depression (13).

In this case, we found that the diagnosis of NPSLE was difficult. When mental disorder was the main clinical symptom, the imaging results were often normal or mildly damaged. Childbearing might be an important factor that induced the aggravation of this disease, as SLE exacerbations generally occurred during the period of late pregnancy or post-parturition. If cicatricial alopecia occurs in women of childbearing age, the pathogenesis needs to be further investigated. The early diagnosis and early treatment of this disease not only reduce the visceral damage but also prevent permanent baldness and subsequent psychological problems.

## Data Availability Statement

The original contributions presented in the study are included in the article/supplementary material, further inquiries can be directed to the corresponding author/s.

## Ethics Statement

Ethical review and approval was not required for the study on human participants in accordance with the local legislation and institutional requirements. Written informed consent was obtained from the patient for the publication of any potentially identifiable images or data included in this article.

## Author Contributions

XH was involved in drafting the manuscript. J-SL revised it critically for important intellectual content and conception and design. All authors given final approval of the version to be published.

## Conflict of Interest

The authors declare that the research was conducted in the absence of any commercial or financial relationships that could be construed as a potential conflict of interest.

## Publisher's Note

All claims expressed in this article are solely those of the authors and do not necessarily represent those of their affiliated organizations, or those of the publisher, the editors and the reviewers. Any product that may be evaluated in this article, or claim that may be made by its manufacturer, is not guaranteed or endorsed by the publisher.
